# Comparison of the effect of combined administration of intravenous and intra-articular tranexamic acid versus their administration alone in the management of blood loss in total knee arthroplasty: a prospective, multicenter study in Iran

**DOI:** 10.1186/s12891-023-07089-z

**Published:** 2023-12-16

**Authors:** Mohammadali Fakharian, Arman Fakharian, Zahra Keshmiri, Amir Mohsen Khorrami

**Affiliations:** 1 Orthopedic Surgeon Department, Mostafa Khomeni Hospital, Shahed University of Medical Sciences, Tehran, Iran; 2 University of California, Davis School of Medicine, Sacramento, CA USA; 3 Shahed University of Medical Sciences, Tehran, Iran; 4 Bone and Joint Reconstruction Research Center, Shafa Orthopedic Hospital, Iran University of Medical Sciences, Baharestan Square, Tehran, 1157637131 Iran

**Keywords:** Total knee arthroplasty, Tranexamic acid, Hemoglobin, Blood transfusion, Blood loss, Intravenous, Intra-articular

## Abstract

**Background:**

Total knee arthroplasty (TKA) is associated with significant blood loss. Antifibrinolytic agents such as tranexamic acid (TXA) are widely used to manage blood loss during TKA. This study aimed to compare the efficacy of three different administration approaches of TXA in TKA.

**Methods:**

In a prospective, multicenter study, 285 patients with end-stage osteoarthritis who underwent TKA between 2020 and 2022 in three orthopedic surgery centers were included in the study. To manage bleeding during TKA, one of the three methods of intravenous administration (IV), intra-articular injection (IA), and combination administration of TXA was performed for the patients. Postoperative blood loss was calculated using blood volume and change in hemoglobin level from preoperative measurement to postoperative day 3.

**Results:**

The mean baseline Hemoglobin (Hb) was not significantly different between the three study groups (*p* > 0.05). The mean postoperative Hb of 12 h, 24 h, and 48 h after the surgery was not significantly different between the three stud groups (*p* > 0.05). The mean intraoperative blood loss in the combined TXA group was significantly lower compared to the IV and IA groups (0.025). The number of blood transfusions in the three study groups was not statistically significant (*p* > 0.05). No side effect was recorded in any group, as well.

**Conclusion:**

Blood loss in the combination TXA group was significantly less than in the other two groups. Combination TXA can help reduce blood loss after TKA surgery.

## Introduction

Osteoarthritis (OA) is the most common degenerative joint disease that affects the daily life of nearly 240 million people (10% of men and 18% of women) worldwide, the majority of whom are elderly [1–3]. Total knee arthroplasty (TKA) is a productive and cost-effective surgery for the treatment of end-stage knee OA [4, 5]. A successful TKA surgery greatly improves the patient’s health-related quality of life [5]. Painless knee movements allow returning to daily activities with no limitations [5].

It has been reported that the mean amount of blood loss during and after TKA ranges between 1400 and 1800 ml so that up to 40% of patients who undergo TKA will require blood transfusion [6, 7], which carries a potential risk of immune reactions and infection transmission [8]. It also imposes a financial burden on both the patients and health systems [9].

Antifibrinolytic agents have been proposed as a part of the blood management protocol in TKA [10]. TXA is a synthetic lysine-analog antifibrinolytic agent that binds to the lysine-binding sites of plasminogen, thereby competitively inhibiting the activation of plasminogen and fibrinolysis [11]. This drug is comprehensively used to manage blood loss in various surgical procedures, including cardiac, gastrointestinal, gynecologic, and neurosurgery [12]. Other drugs, such as intraarticular epsilon aminocaproic acid (EACA) can be used as an alternative to TE to control bleeding. In the EJPF study, Guerreiro et al. showed that TXA and EACA similarly reduced Hgb and Htb in the first 48 h after TKA [13].

There are some concerns that TXA might provoke thromboembolic events [14]. However, preliminary studies have confirmed the efficacy of TXA in reducing blood loss after TKA with no increased risk of deep venous thrombosis and thromboembolism [15–18]. JPF Guerreiro et al. [19] reported that, in addition to reducing bleeding, topical TXA can improve pain and increase flexion in the early hours after TKA.

Therefore, its implication in TKA is safe, effective, and cost-efficient [17]. TXA could be administered in different routes, including IV, IA, and a combination. Despite numerous studies on this subject, the optimal route for TXA administration remains controversial [20–24]. The previous studies comparing the efficacy of administration routes (IV vs. IA) concluded that they are similarly efficient [25].

The duration of joint exposure to TXA varies in different approaches, which can affect absorption quality. Therefore, in this study, we aimed to compare the effectiveness of three TXA administration methods (IV, IA, and combined IV and IA regimens) in managing blood loss in patients undergoing TKA.

## Patients and methods

### Patients and setting

This study was approved by the ethics committee of Shahed University of Medical Sciences with code: IR.Shahed.REC.1396.29. In this prospective, multi-center cohort study, 456 patients with end-stage osteoarthritis who underwent TKA surgery in three hospitals in Tehran (Mostafa Khomeini Hospital, Moheb Kowsar Hospital, and Parsian Hospital) between 2020 and 2022 were examined. Routinely, during TKA, TXA was used to manage bleeding with one of the three methods of intravenous infusion, intra-articular washing, or intravenous infusion and intra-articular washing. The researcher had no intervention in choosing the treatment method and surgery of the patients. Two hundred eighty-five patients were included in the study. Patients were divided into three groups based on the type of TXA method used to manage blood loss.

The first group included 108 patients who received an IV infusion of TXA (1 g in 100 cc of normal saline) before the tourniquet. The second group included 92 patients who underwent IA washing with TXA (2 g in 75 ccs normal saline) for five minutes before closing the arthrotomy. After five minutes, TXA and normal saline solution were suctioned. The third group consisted of 86 patients with intravenous infusion and intra-articular washing. All patients were operated on by a surgeon with more than ten years of experience. Two prosthesis types, cruciate retaining (CR) and posterior stabilized (PS), were used. According to the surgeon’s opinion, according to the guidelines of the American Hip and Knee Association (AAHKS) [26], all patients received TNX routinely unless they had contraindications (MI, recent thromboembolic events, etc.), and these patients were excluded from the study.

Tourniquets have been used routinely for all patients. The tourniquet was placed before cutting the skin. Then, at the end of the procedure, it was removed after the skin was completely sewn. All patients were hospitalized 24 h after the surgery under specialist supervision. Patients were NPO two hours after surgery. Routinely, in the first 24 h after surgery, all patients received about 2 L of normal saline or half saline or Ringer’s serum according to their conditions.

### Inclusion and exclusion criteria

The inclusion criteria for the study included patients with end-stage osteoarthritis who are candidates for TKA and access to the laboratory profile of the patients. Acquired color vision deficiency, coagulation problems (platelet count < 150/µl, INR < 1.4 and other coagulation or bleeding disorders), vascular disorders, cerebral disorders (stroke), cardiovascular disorders (history of myocardial infarction, shunting, coronary insufficiency class 3, coronary insufficiency 3, heart failure 3), embolic events (deep vein thrombosis, pulmonary embolism), sensitivity to TXA and history of seizure disorders were defined as exclusion criteria.

TKA was performed for all patients under similar conditions.

### Data collection

Demographic characteristics (age, gender, body mass index (BMI), smoking, and comorbidities) and findings during and after surgery (duration of surgery, need for blood transfusion, hemoglobin (Hb) level before surgery, Hb level 12 h after surgery, Hb level 24 h after surgery and Hb level 3 day after surgery, total blood loss three days after surgery) were collected and recorded by the researcher using a checklist. Hb < 8 mg/dl or Hb = 8-10 mg/dl with clinical symptoms or signs of significant blood loss such as dizziness, tachycardia, shortness of breath, palpitations, or signs of organ failure due to blood loss) was defined as the need for blood transfusion before, during, and after surgery [27]. Findings were compared in different periods in three groups. To control the confounding variables among the three groups, Frequency Matching was performed for the variables of age, gender, body mass index, smoking, and comorbidities with the opinion of the epidemiologist.

### Total blood loss measurement

Blood loss after surgery was calculated using the blood volume, and the hemoglobin change was calculated from before the operation to 48 h and day three after the operation [28]. To estimate the patient’s blood volume, we used the formula introduced by Nadler et al. [29]. Total blood loss was estimated for all three approaches three days after surgery.

### Sample size collection

The appropriate sample size for conducting this study, according to the estimation of the difference of 437 ml reduction in postoperative blood loss in the comparison of different regimens of TXA administration for total knee arthroplasty, based on the study of S Tsukada et al. [30] with an effect size of 0.33, an alpha error of 0.05 and a power of 80%, was estimated using G Power version 3.1 software, the number of 37 patients for each group.

### Statistical analysis

Data were analyzed using SPSS version 22 software. Qualitative parameters were reported with descriptive statistics (frequency and %). Mean and standard deviation were used to report quantitative variables. The Shapiro-Wilk test was used to evaluate the normality of the distribution of the variables. When comparing quantitative variables between two groups, the independent t-test is used if the variable distribution is normal. If the distribution is not normal, the non-parametric Mann-Whitney test is used. The ANOVA test was used under the normality assumption to compare the variables in more than two groups, and the Kruskal–Wallis test was used in case of abnormality. Repeated measures ANOVA test was used to compare the level of Hb at different times in three groups. A *p*-value less than 0.05 was considered a statistical significance level.

## Results

The overall mean age was 69.55 ± 8.8 years. 252(88.4%)% of patients were women. Hypertension was the most common comorbidities in patients. The prevalence of hypertension in intravenous, intra-articular, and combined groups was 24.06%, 25%, and 23.3%, respectively (p:0.33). PS-type prosthesis was used in 178 (57.3%) cases. No statistically significant difference was observed for the demographic characteristics of the patients in the three groups (Table 1).

**Table 1:** Demographic characteristics of patients who underwent TKA

Variable	Group			*P*-value
	IV group (*n* = 108)	IA group (*n* = 92)	Combined group (*n* = 86)	
Gender				0.53
• Male	15(13.9%)	9(9.7%)	9(10.5%)	
• Female	93(86.1%)	83(90.3%)	77(89.5%)	
Age (year)	69.21 ± 8.9	70.01 ± 7.6	68.55 ± 8.22	0.46
Body mass index (Kg/m^2^)	28.25 ± 4.1	28.5 ± 3.88	27.56 ± 4.55	0.52
Diabetes mellitus				0.26
• Yes	19 (17.6%)	15 (16.3%%)	13 (15.1%)	
• No	89 (82.4%)	77 (83.7%)	73 (84.9%)	
Hypertension				0.33
• Yes	26 (24.07%)	23 (25%%)	20 (23.3%)	
• No	82 (75.93%)	69 (75%)	66 (76.7%)	
Smoking				0.28
• Yes	27 (25%)	26 (26.1%%)	23(26.7%)	
• No	81 (75%)	66(73.9%)	63 (73.3%)	
Prosthesis type				0.88
• CR	61(56.5%)	54(58.7%)	49(57%)	
• PS	47(43.5%)	38(41.3%)	37(43%)	

*IV* Intravenous, *IA* Intra-articular, *CR* Cruciate retaining, *PS* Posterior stabilized

Assuming normality, ANOVA test was used and in case of non-normality, Kruskal–Wallis test was used to compare the variables in three groups

Four units of packed red blood cells were transfused: two in the IV, one in IA, and one in the combined group. None of the patients in the intra-articular group required a blood transfusion. Based on the chi-squared test, the difference in the number of blood transfusions in the three study groups was not statistically significant (p:0.22). The mean duration of surgery in intravenous intra-articular and combined groups was 65.1 ± 9.9, 63.5 ± 6.4, and 64.8 ± 7.9 min, respectively, which this difference was not statistically significant (p:0.38).

The mean Hb of the day before the surgery, 12 h after the surgery, and 24 h after the surgery are demonstrated in Table 2. The mean preoperative Hb significantly differed from the postoperative Hb of 12 and 24 h after the surgeries in all groups (*p* < 0.05). According to ANOVA tests, the mean preoperative Hb of the three groups did not show a significant difference (*p* > 0.05). No significant difference was observed for the mean Hb 24 h after surgery (*p* < 0.05). No significant difference was observed for Hb level and mean total blood loss in both IV and IA groups (*p* < 0.05). The mean three days after the TKA in the combined TXA group was higher than the other two groups (p:0.025). The mean intraoperative blood loss in the combined TXA group was significantly lower compared to the IV and IA groups (P:0.018). No TXA-related side effect was recorded in any groups of the study.

**Table 2:** Comparison of the mean Hb between the three study groups at three time-points

Time-point	Group			*P*-value
	IV group (*n* = 108)	IA group (*n* = 92)	Combined group (*n* = 86)	
Preoperative Hb (mg/dl)	13.21 ± 1.4	13.5 ± 1.5	13.48 ± 1.3	0.89
Hb of 12 h after the surgery(mg/dl)	11.95 ± 1.5	11.84 ± 1.6	12.01 ± 1.5	0.88
Hb of 24 h after the surgery	10.53 ± 1.7	10.49 ± 1.6	10.67 ± 1.4	0.28
Hb of 3 days after surgery (mg/dl)	9.21 ± 1.8	9.58 ± 2.68	11.08 ± 1.28	0.001
Total blood loss (ml)	1611.2 ± 401.1	1601.6 ± 392.2	1256.4 ± 331.1	0.025

*Hb* Hemoglobin; *IV* Intravenous; *IA* Intra-articular

Repeated measures ANOVA test was used to compare the level of variables at different times in three groups

## Discussion

TXA is an antifibrinolytic agent that effectively reduces blood loss in TKA surgeries. Due to its availability and safety, it is widely used with no concern. In TKA, both IV and IA administrations of TXA are commonly used. Although several studies have been performed on this subject, the results often must be more consistent. This inconsistency could be attributed to a variety of factors. Despite the efficacy of TXA in managing blood loss in TKA, the optimal dose of the drug is still controversial. The duration of joint exposure to TXA varies in different approaches. In this study, we evaluated the effectiveness of three TXA administration approaches (IV, IA, and combined IV and IA regimens) in managing blood loss in patients who underwent TKA. The results of our study showed that all three approaches were effective in managing blood loss. No significant difference between the three groups was observed for hemoglobin levels in three periods before surgery and 12 and 24 h after surgery. No significant difference was observed in the need for blood transfusion and the number of injected blood packs in the three groups. Also, in terms of the mean duration of surgery, there was no significant difference between the three groups. No significant difference was observed for Hb level and mean blood loss in both IV and IA groups. The mean intraoperative blood loss in the combined TXA group was significantly lower compared to the IV and IA groups, which were consistent with the results of the studies [25, 27, 31].

S Tsukada et al. [30] by evaluating the effect of combined IV and IA TXA in controlling blood loss after surgery in simultaneous bilateral total knee arthroplasty without the use of tourniquets on 30 patients, showed that in the combined TXA group, the mean blood loss during and postoperative was significantly lower than in the IV TXA group. They also showed in their study that no patient in the combined TXA group required a blood transfusion. No case of heart attack or thrombotic event was reported in the treatment group, which confirmed the results of our study. In line with the results of our study, YM Zhang et al., by evaluating the combined effect of IV and IA TXA in TKA to prevent blood loss compared to the effect of IV and IA, showed that combined IV plus IA TXA injection was significantly associated with reducing blood loss in TKA. In 2022, in a systematic review and meta-analysis, T Ling et al. [32] evaluated the efficacy and safety of combined IV and IA TXA administration in TKA. They showed that the combined administration of IV and IA TXA was significantly more effective in reducing total blood loss, transfusion rate, postoperative hemoglobin drop, and drainage output after TKA than the effect of a single IV and TV, which confirmed the results of our study. In another study, in 2023, C Zheng et al. [33] showed that the combined administration of IV plus AI significantly reduced blood loss compared to the administration of IV and IA alone in TKA. While in a study by Y Dorji et al. [34] evaluated the effectiveness of the combined use of IV and IA TXA versus their single effect in reducing blood loss in primary total knee arthroplasty in a randomized controlled clinical trial study on 31 patients; they did not report a significant difference in reducing blood loss in the combined TXA group compared to their single group in TKA. The difference in the results can be justified due to the difference in the sample size of the investigated patients in the two groups.

In a double-blind, randomized, non-inferiority clinical trial, Goyal et al. compared the intravenous (IV) and intra-articular (IA) administration of TXA in patients with their primary unilateral TKA. According to their report, no significant difference was found between the blood loss on the first postoperative day between the IV and IA administration of TXA [35]. Keyhani et al. also compared the efficacy of IV and IA TXA administration in reducing blood loss and transfusion rate in patients who underwent primary TKA. Their results showed that the IV and IA routes did not differ significantly regarding postoperative blood loss and transfusion rate. No thromboembolic event was recorded in this study [31].

A comparison of the TXA administration route in TKA patients has also been performed in several other investigations. A meta-analysis of the randomized clinical trials and prospective cohorts performed by Wang et al. [36] revealed no significant difference between IV and IA administration TXA regarding their effect on reducing blood loss and transfusion rate in TKA.

Hamlin et al. [22] used a TXA dose 1gr for IV administration and 3gr for IA administration. They reported more efficacy of IA administration of TXA in reducing blood loss in TKA patients. Yuan et al. [21], in a systematic review of 10 clinical trials, evaluated the efficacy of combined IV/IA administration of TXA in reducing blood loss in TKA patients. They concluded that the efficacy of combined administration is superior to the efficacy of each administration alone. The same results were reported in the study of Jain et al. [15] and Xie et al. [20].

Our study had strengths and weaknesses that should be mentioned. Due to the study’s design, we could not compare the functional outcomes of the knee in the studied groups, which was the most important weakness of our study. Also, due to the lack of design of the study as a randomized controlled clinical trial, the characteristics of the patients in the groups may affect the study’s results to some extent. However, we tried to reduce the random error as much as possible by applying matching and examining a large sample size of patients in the subgroups. The most important strength of this study was designing a comprehensive study with a suitable sample size to evaluate the effect of combined TXA administration on reducing blood loss and comparing it with single groups.

## Conclusion

Our study showed that the blood loss in the combined IV/IA TXA group was significantly less than in the IV and IA groups. A TXA combination can be prescribed as a safe method in TKA to reduce blood loss after TKA surgery and accelerate patients’ rehabilitation process after surgery.

## Data Availability

The datasets used and/or analysed during the current study are available from the corresponding author on reasonable request.

## Abbreviations

TKA: Total knee arthroplasty
TXA: Tranexamic acid
IV: Intravenous administration
IA: Intra-articular injection
HB: Hemoglobin (Hb)
